# Immunotherapy of COVID-19: Inside and Beyond IL-6 Signalling

**DOI:** 10.3389/fimmu.2022.795315

**Published:** 2022-02-22

**Authors:** Gaetano Zizzo, Antonio Tamburello, Laura Castelnovo, Antonella Laria, Nicola Mumoli, Paola Maria Faggioli, Ilario Stefani, Antonino Mazzone

**Affiliations:** Department of Internal Medicine, Azienda Socio Sanitaria Territoriale (ASST) Ovest Milanese, Milan, Italy

**Keywords:** COVID-19, cytokines, IL-6, therapy, tocilizumab, sarilumab, baricitinib, anakinra

## Abstract

Acting on the cytokine cascade is key to preventing disease progression and death in hospitalised patients with COVID-19. Among anti-cytokine therapies, interleukin (IL)-6 inhibitors have been the most used and studied since the beginning of the pandemic. Going through previous observational studies, subsequent randomised controlled trials, and meta-analyses, we focused on the baseline characteristics of the patients recruited, identifying the most favourable features in the light of positive or negative study outcomes; taking into account the biological significance and predictivity of IL-6 and other biomarkers according to specific thresholds, we ultimately attempted to delineate precise windows for therapeutic intervention. By stimulating scavenger macrophages and T-cell responsivity, IL-6 seems protective against viral replication during asymptomatic infection; still protective on early tissue damage by modulating the release of granzymes and lymphokines in mild-moderate disease; importantly pathogenic in severe disease by inducing the proinflammatory activation of immune and endothelial cells (through trans-signalling and trans-presentation); and again protective in critical disease by exerting homeostatic roles for tissue repair (through cis-signalling), while IL-1 still drives hyperinflammation. IL-6 inhibitors, particularly anti-IL-6R monoclonal antibodies (e.g., tocilizumab, sarilumab), are effective in severe disease, characterised by baseline IL-6 concentrations ranging from 35 to 90 ng/mL (reached in the circulation within 6 days of hospital admission), a ratio of partial pressure arterial oxygen (PaO2) and fraction of inspired oxygen (FiO2) between 100 and 200 mmHg, requirement of high-flow oxygen or non-invasive ventilation, C-reactive protein levels between 120 and 160 mg/L, ferritin levels between 800 and 1600 ng/mL, D-dimer levels between 750 and 3000 ng/mL, and lactate dehydrogenase levels between 350 and 500 U/L. Granulocyte-macrophage colony-stimulating factor inhibitors might have similar windows of opportunity but different age preferences compared to IL-6 inhibitors (over or under 70 years old, respectively). Janus kinase inhibitors (e.g., baricitinib) may also be effective in moderate disease, whereas IL-1 inhibitors (e.g., anakinra) may also be effective in critical disease. Correct use of biologics based on therapeutic windows is essential for successful outcomes and could inform future new trials with more appropriate recruiting criteria.

## Introduction

As of October 1, 2021, the Coronavirus Disease 2019 (COVID-19) pandemic has caused over 200,000,000 cases and more than 4,500,000 deaths (1). Although severe acute respiratory syndrome coronavirus 2 (SARS-CoV-2) infection is more often asymptomatic, on the other hand healthy carriers importantly contribute to the spread of the virus, and COVID-19 can manifest itself in different forms of severity, namely mild, moderate, severe and critical.

Mild forms affect patients with respiratory symptoms who are generally not hospitalised and do not require supplemental oxygen. Moderate forms affect patients with viral pneumonia who require low-flow supplemental oxygen (LFO, ≤ 5 liters per minute). Severe forms affect patients with bilateral interstitial pneumonia and acute respiratory distress syndrome (ARDS) requiring high-flow oxygen (HFO) or non-invasive ventilation (NIV). Critical forms affect patients admitted to intensive care unit (ICU) with severe ARDS, shock, and/or multiple organ failure, requiring invasive mechanical ventilation (IMV) with or without other organ support therapies, such as vasopressors, extracorporeal membrane oxygenation (ECMO), or dyalisis (2).

Severe and critical forms represent 14-15% and 2-5% of cases, respectively (3–6). Such life-threatening conditions are believed to result from a SARS-CoV-2-induced respiratory and systemic autoinflammatory disease, in which a dysregulated immune response, associated with exuberant cytokine release, would ultimately account for widespread vascular and tissue damage (7). Cytokines play a central role in the pathogenesis of COVID-19, emerging both as useful biomarkers in predicting disease evolution and as strategic targets for therapy. Discrete clusters of cytokines and chemokines are differentially expressed according to disease stage, with molecules involved in lymphoid priming being upregulated in moderate disease, and molecules involved in myeloid differentiation and migration being overexpressed in severe disease. A deep understanding of the qualitative, quantitative and temporal differences in cytokine pathways is therefore essential to appropriately choosing the right drug and the right timing for effective treatment (7, 8). While direct antiviral therapies may be useful in the early stages of the disease to inhibit virus replication, immunomodulatory and anti-cytokine therapies aimed at targeting harmful hyperinflammation would be crucial in the overt stages, to avoid critical deterioration and fatal outcomes. Since mortality rates can exceed 80% in critically-ill patients requiring IMV (9, 10), acting in severe progressive forms appears to be of the utmost importance in reducing lethality.

## IL-6 in COVID-19 Inflammatory Cascade: Rationale for a Key Role in Severe Disease Progression

IL-6 levels in the circulation and bronchoalveolar lavage fluid of patients with COVID-19 progressively increase with disease severity (11, 12), reaching their peaks in critically-ill patients (13, 14). In the lungs of COVID-19 patients, IL-6 can be released by SARS-CoV-2 infected respiratory epithelial cells (15, 16), as well as by infiltrating CD14^+^CD16^+^ monocytes-macrophages and CD4^+^ T cells (17).

IL-6 can substantially contribute to the dysregulation of the immune response in COVID-19, basically by acting in two directions: on one side, it may cause a dysfunction of natural killer and cytotoxic CD8^+^ T cells associated with perforin and granzyme down-regulation (18), thereby dampening antiviral defenses; on the other side, it may inhibit the differentiation of regulatory T cells and elicit a T helper 17 (TH17)-like polarization of γ/δ and α/β CD4^+^ T cells, thus leading to uncontrolled hyperinflammation (7). At advanced stages, these mechanisms would result into a macrophage activation syndrome (MAS)-like condition, characterised by lymphocyte exhaustion and aberrant innate immune responses, vascular leakage, exudative-phase ARDS, coagulopathy, and multi-organ failure (7, 19). In severe COVID-19, in fact, increased levels of IL-6 have been found to be associated with higher viral load (20), lymphopenia and neutrophilia (11, 21), systemic inflammation (22), hypoxemia (23), and poor prognosis (22, 23). On the other hand, certain polymorphisms in the IL-6 receptor gene that attenuate IL-6 signalling have been shown to be protective against disease progression, resulting in a lower risk of hospitalisation for COVID-19 (24). Therapeutic blockade of IL-6 may therefore represent an effective strategy to prevent worsening of respiratory status and reduce overall mortality in these patients.

## Inhibitors of IL-6 Signal in COVID-19: What to Hit and When?

**Figure 1:**
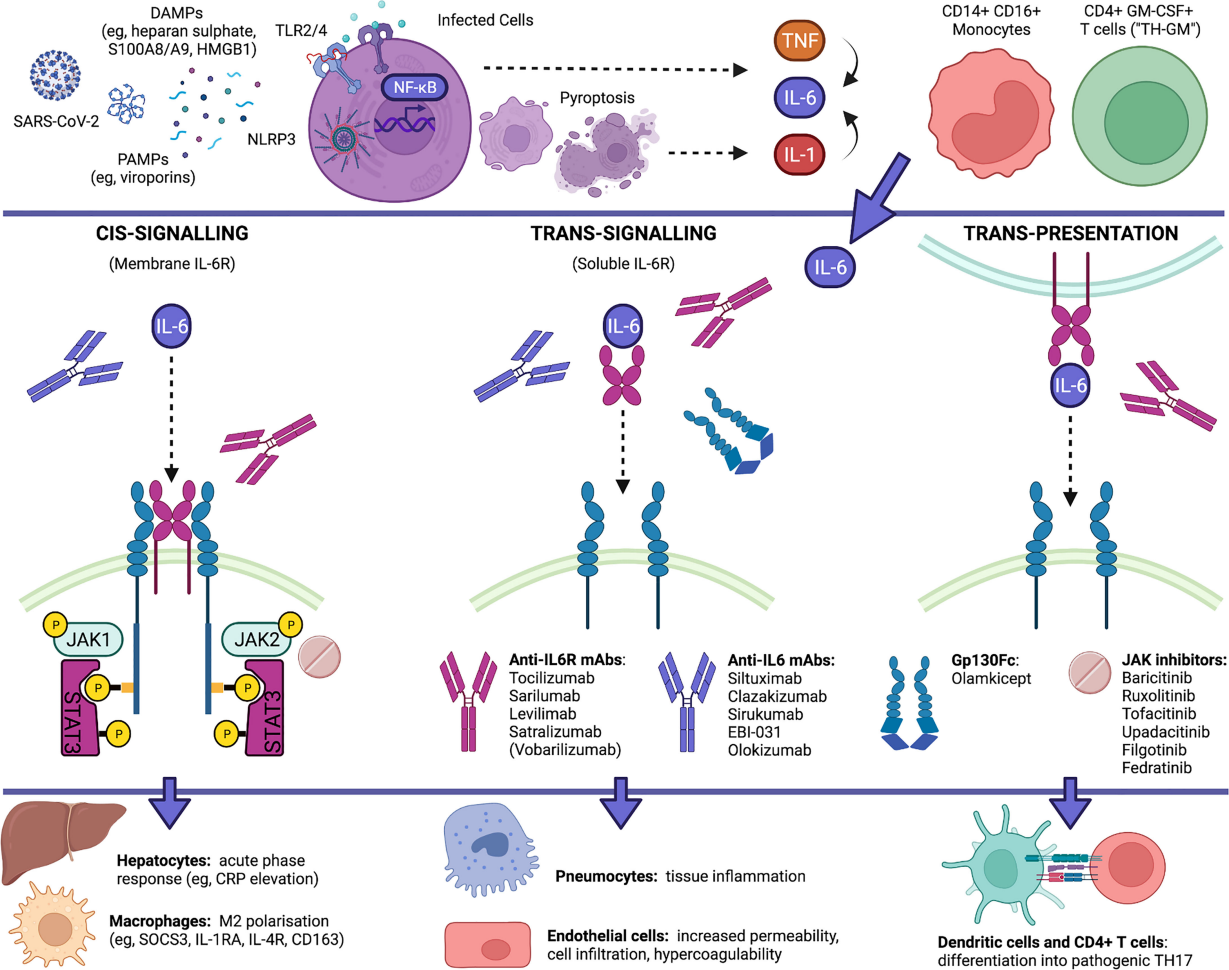
IL-6 pathways in COVID-19 and currently available blockers of IL-6 signalling. IL-6 is increasingly upregulated in COVID-19, being released by SARS-CoV-2 infected cells as well as by proinflammatory and infiltrating monocytes and T cells. IL-6 production is placed downstream of an autoinflammatory loop linked to pyroptosis and IL-1 production that is elicited by virus-associated PAMPs (e.g., viroporins) *via* the NLRP3 inflammasome and downstream of the activation of membrane TLRs by virus-induced DAMPs (e.g., heparan sulphate, alarmins S100A8/A9 and HMGB1), and occurs in parallel with the release of other cytokines (e.g., TNF, IL-10, IL-8, CCL2). IL-6 can signal through at least three distinct modalities. Anti-IL-6R antibodies, anti-IL-6 antibodies, gp130Fc and JAK inhibitors can differentially inhibit IL-6 signalling by acting at different sites. Whereas IL-6 trans-signalling and trans-presentation enhance the proinflammatory activation of pneumocytes, endothelial cells and T cells, IL-6 cis-signalling exerts homeostatic roles by eliciting the clearance of remnants through hepatic release of opsonins and the differentiation of scavenger macrophages. Created with Biorender.com.

IL-6 is a pleiotropic cytokine with multiple functions and a complex signalling involving two receptor subunits: IL-6Rα (IL-6R) and IL-6Rβ (130-kDa glycoprotein or gp130). Several signalling modalities have been identified, namely: cis-signalling (or classic mode), in which IL-6 binds to membrane IL-6R (e.g., on macrophages, hepatocytes, megacaryocytes, intestinal epithelial cells) and gp130; trans-signalling, in which a complex formed by IL-6 and soluble IL-6R (mainly cleaved by a disintegrin and metalloprotease-17 or ADAM-17) binds to membrane gp130 (e.g., in endothelial cells, neutrophils, monocytes, pneumocytes); and trans-presentation, in which IL-6 first binds to IL-6R on the membrane of one cell (e.g., dendritic cells) and then the complex binds to gp130 in another cell (e.g., CD4^+^ T cells) (25–27). Different modes result in different effects: cis-signalling mediates host protection against intracellular pathogens and tissue homeostasis, by upregulating opsonins in the liver and by inducing scavenger and regulatory functions in macrophages; trans-signalling drives proinflammatory activation of pneumocytes, adipose tissue-associated macrophages, gut-associated immune cells, neutrophils and endothelial cells; while trans-presentation promotes T-cell differentiation into pathogenic TH17 cells (25, 26) (**Figure 1**).

IL-6 trans-signalling and trans-presentation are most likely pathogenic in severe progressive COVID-19: through trans-presentation, IL-6 may act, *via* signal transducer and activator of transcription (STAT) 3, to polarize granulocyte-macrophage colony-stimulating factor (GM-CSF)-producing T cells (“TH-GM”), induced by IL-7, IL-23 and IL-1, into full-blown TH17 cells, thus amplifying neutrophil recruitment and activation (7); through trans-signalling, IL-6 may further contribute to upregulate chemokines (e.g., CCL2, IL-8), adhesion molecules (e.g., intercellular adhesion molecule-1, while internalizing vascular endothelial cadherin), their ligands (e.g., CD11b/CD18 integrin), and procoagulant factors (e.g., tissue factor-1, factor-III), thereby leading to increased endothelial permeability, CD14^+^CD16^+^ (intermediate/non-classical) monocyte and neutrophil migration from peripheral blood into inflamed tissues, formation of neutrophil extracellular traps (NETs), neutrophil-endothelial cell interactions, and hypercoagulability (7, 12, 17, 28–30). IL-6 trans-signalling and trans-presentation may therefore account for diffuse inflammation at various levels, including pulmonary, vascular, intestinal, and obesity-enhanced inflammation, up to the condition of shock, secondary to cytokine-mediated multi-organ dysfunction, vascular leakage and microthrombosis.

Whereas the soluble decoy receptor sgp130Fc (e.g., olamkicept) selectively blocks trans-signalling, anti-IL-6 monoclonal antibodies (e.g., siltuximab, clazakizumab, olokizumab) can block cis-signalling and trans-signalling, and anti-IL-6R monoclonal antibodies (e.g., tocilizumab, sarilumab) can block all three mechanisms, including trans-presentation (25). By acting on soluble IL-6R, monoclonal antibodies might also affect the signalling of IL-27p28/IL-30, possibly interfering with the induction of CXCL10 (31), another chemokine that is strongly upregulated in COVID-19 (7). Since tocilizumab, but not siltuximab, has been shown from meta-analyses to be effective in patients hospitalised with COVID-19 (32), blocking trans-presentation and IL-6-induced TH17 polarization would be particularly crucial to prevent severe to critical disease progression.

Conversely, IL-6 cis-signalling mainly drives hepatic synthesis and secretion of acute-phase reactants (e.g., CRP elevation in the blood), and exerts negative feedback mechanisms on proinflammatory cytokines, by suppressing their production (e.g., TNF) (33), stimulating their decoy receptors (e.g., sTNFRp55, IL-1RA) (34), or inducing negative regulators of their intracellular pathways (e.g., suppressor of cytokine signalling 3, or SOCS3, inhibiting IL-6, IL-7 and IL-23 signals; and IL-4R, inhibiting TH17 maturation) (7, 25, 26, 35). In the circulation of patients with COVID-19, IL-6 reaches its peak at advanced stages, on average after the second week of disease, and, notably, this increase is accompanied by peak concentrations of IL-10 and CRP (11, 13). This suggests that in critical COVID-19, strong elevation of IL-6 (e.g., > 100-120 pg/mL) and CRP levels (e.g., > 160-200 mg/L) could reflect augmented IL-6 cis-signalling in the attempt to exert homeostatic roles, while IL-10 further strengthens the predominance of SOCS3 over STAT3 signal (36). Therefore, inhibiting membrane IL-6R at this stage would not be useful (and might even worsen inflammation), as indicated by poor outcomes with tocilizumab in critically-ill patients. Furthermore, since IL-6 cis-signalling elicits host defence against pathogens and promotes the growth and survival of hepatocytes, megacaryocytes and intestinal epithelial cells (25, 26), membrane IL-6R blockade might promote adverse events and complications, including serious infections (e.g., ventilator-associated pneumonia) (37, 38), increased transaminases, thrombocytopenia and intestinal perforation, particularly in patients who are critically-ill with COVID-19, as they are intubated, immunosuppressed, and inflamed or hypoperfused at multiple levels.

In early stages of COVID-19, in which IL-6 may play protective roles, IL-6R blockade would not be useful either. Many viruses, in fact, including SARS-CoV-2, are able to induce SOCS3 as a virulence factor, which inhibits STAT1/IFN and gp130/JAK2/STAT3 signals to elude antiviral responses and virus clearance, thus allowing viral replication (39–41); collaterally, STAT3 down-modulation results in a compensatory induction of IL-6 mediated by nuclear factor kappa-light-chain-enhancer of activated B cells (NF-κB) (41), thereby already mimicking the effects of anti-IL-6R agents. Moreover, viral pneumonia in mild-moderate COVID-19 might be substantially mediated by cytokines and cellular pathways pathogenically upstream of IL-6, such as IL-33, IL-9 or IFNγ, which can be released by virus-damaged respiratory epithelial cells, type-2 innate lymphoid cells and γ/δ T cells (7); indeed, at this stage, IL-6 may negatively modulate T-cell release of IFNγ and granzymes (18, 42).

In fact, tocilizumab was observed to reduce IL-17 and TNF (i.e., cytokines associated with severe disease), yet it provoked a transitory increase in IFNγ (probably associated with moderate disease), and a strong increase in IL-6 and IL-10 levels (highly associated with critical disease) (43).

**Figure 2:**
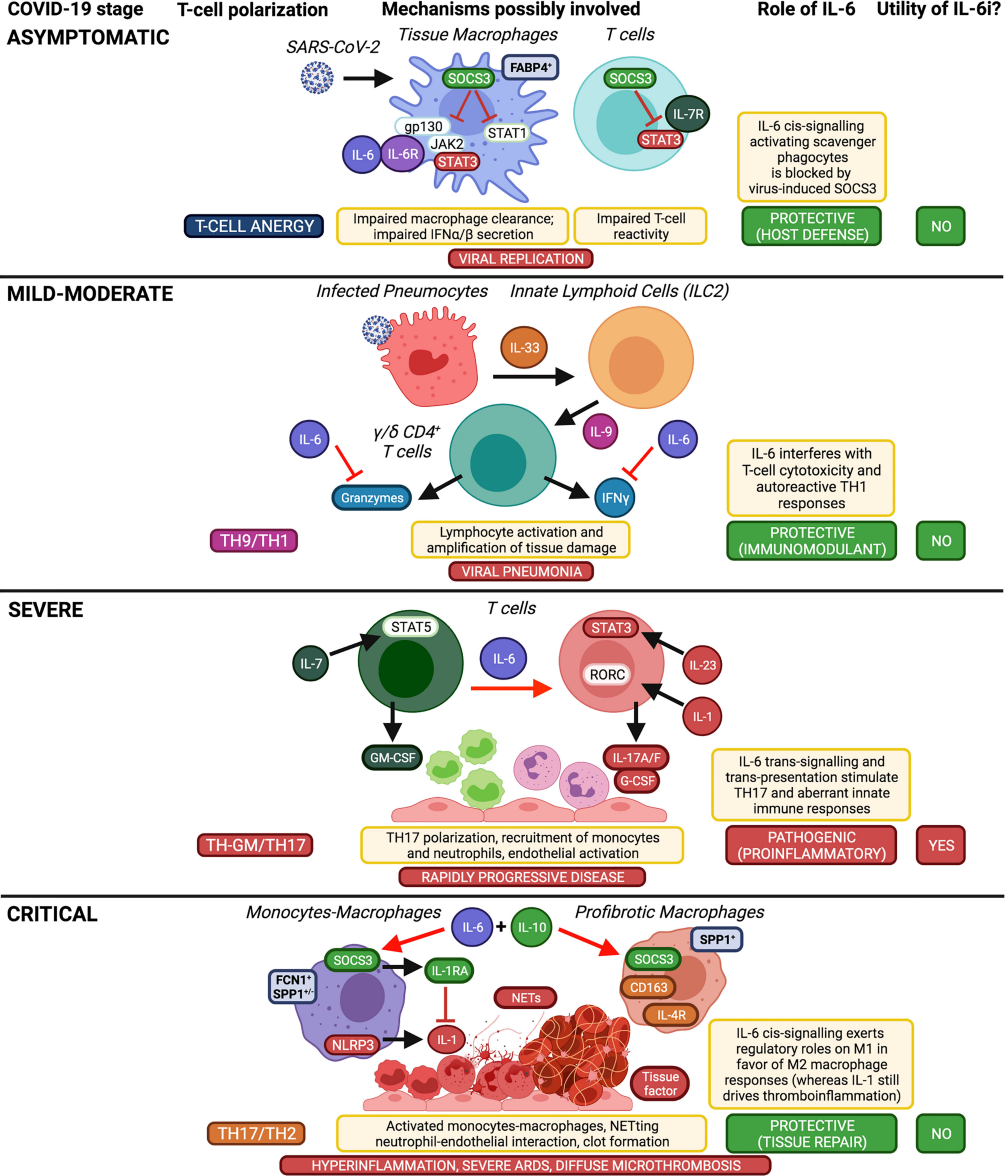
Implications of IL-6 in COVID-19 pathogenesis and therapeutic opportunity of using IL-6 inhibitors according to disease stage. Taking into account the well-established functions of IL-6 on immune cells (i.e. pro-M2, anti-Treg, anti-cytotoxic, anti-TH1, pro-TH17), here we schematically summarize the differential roles of IL-6 in the inflammatory cascade of COVID-19 according to disease stages and supposed T-cell polarization, which affect the usefulness or otherwise of IL-6 inhibitors (IL-6i). Created with Biorender.com.

Taken together, benefits from therapeutic blockade of IL-6 can be obtained in COVID-19 by disrupting proinflammatory IL-6 trans-presentation and trans-signalling, which would be predominant and pathogenic in severe disease; anti-IL-6 treatment should therefore be given to patients with severe and rapidly progressive COVID-19, within the second week of symptom onset (or within the first week of hospitalisation), that is before the increased IL-6 and CRP levels further rise uncontrollably. By contrast, IL-6 inhibition no longer appears to be useful in critically-ill patients, in whom IL-6 cis-signalling predominates and would now exert anti-inflammatory and homeostatic roles. IL-6 inhibition does not seem to be useful even in early stages of COVID-19, as IL-6 signalling would be protective, indeed already blocked by the virus, and mild-moderate disease would be primarily driven by viral replication and additional upstream cytokines (**Figure 2**).

## Tocilizumab in Early Observational Studies: What Have We Learned From the Italian Experience?

Tocilizumab is an anti-IL-6R monoclonal antibody that is approved, and widely available in both intravenous and subcutaneous formulations, for certain acute and chronic inflammatory disorders that share many clinical and pathogenic aspects with severe COVID-19, such as autoinflammatory febrile diseases (e.g., Still’s disease), autoimmune diseases that are complicated by interstitial lung disease (e.g., rheumatoid arthritis), vasculitis (e.g., giant cell arteritis), and cytokine storm syndromes (e.g., MAS complicating Still’s disease, chimeric antigen receptor T-cell therapy-induced cytokine release syndrome or CAR-T cell-induced CRS). In these contexts, tocilizumab can efficiently act on fever, fatigue, pain, anxiety and depression, anemia, acute-phase response, deterioration of lung function, and vascular manifestations (44).

Italy was among the first countries after China to face the COVID-19 outbreak. It was soon realised that patients hospitalised with COVID-19 who worsened and died had developed a hyperinflammatory reaction largely responsible for severe ARDS and multi-organ failure. Tocilizumab and other IL-6 inhibitors (i.e., sarilumab, siltuximab) were then tried as compassionate off-label use in an effort to relieve harmful inflammation.

From the literature, we identified 10 retrospective cohort studies, published between May 9, 2020, and January 8, 2021, that enrolled patients hospitalised with COVID-19 during the “first wave” of the outbreak in Italy (overall between the end of February and the end of May, 2020), who underwent treatment with IL-6 inhibitors versus standard of care only (45–54). Studies were somewhat heterogenous in the number of patients recruited or included in the treatment arm, as well as in treatment protocols (including route of administration, dosage, number and timing of infusions), co-treatments, and disease severity (PaO2/FIO2 ratio and magnitude of systemic inflammation) (**Table 1**).

**Table 1:** Early Italian observational cohort studies with IL-6 inhibitors in COVID-19.

PROVED EFFICACY	NO	YES	NO	PARTIAL	YES	YES	NO	YES	PARTIAL	YES
Retrospective Cohort Study [Ref.]	Colaneri et al. (45)	Capra et al. (46)	Quartuccio et al. (47)	Campochiaro et al. (48)	Gritti et al. (49)	Guaraldi et al. (50)	Della Torre et al. (51)	Rossotti et al. (52)	Canziani et al. (53)	Castelnovo et al. (54)
**STUDY CHARACTERISTICS:**										
Date of publication	May 9, 2020	May 13, 2020	May 15, 2020	May 22, 2020	Jun 20, 2020	Jun 24, 2020	Jul 3, 2020	Jul 8, 2020	Jul 8, 2020	Jan 8, 2021
Period of enrollment (2020)	Mar 14 - Mar 27	Feb 26 - Apr 2	Feb 29 - Apr 6	? - Mar 19	Feb 23 - Apr 9	Feb 21 - Apr 30	Mar 14 - Apr 2	Mar 13 - Apr 3	Mar 15 - Apr 22	Mar 6 - May 30
Treatment arm (route, dosage, number of doses)	Tocilizumab IV 8 mg/kg (x2)	Tocilizumab IV 400 mg or SC 324 mg (x1)	Tocilizumab IV 8 mg/Kg (x1)	Tocilizumab IV 400 mg (x1-2)	Siltuximab IV 11 mg/kg (x1-2)	Tocilizumab IV 8 mg/kg (x2) or SC 324 mg (x1)	Sarilumab IV 400 mg (x1)	Tocilizumab IV 8 mg/kg (x1-2)	Tocilizumab IV 8 mg/Kg (x2)	Tocilizumab IV 8 mg/kg (x2-3)
Number of patients recruited	112	85	111	65	60 matched (218 total)	544	56	222	128	112
Number of patients in the treatment arm	**21**	**62**	**42**	**32**	30	**179** (88 iv + 91 sc)	**28**	**74**	**64**	**50**
**PATIENT CHARACTERISTICS (treatment arm):**										
(Median) Age (years old)	62	**63**	62	**64**	**64**	**64**	**56**	**59**	(mean) **73**	(mean) **61**
Male sex (%)	90.5	**73**	79	**91**	**77**	**71**	85	**82**	**63**	**70**
(Median) Number of Days from hospitalization	2	**≤4**					2			**4**
(Median) Number of Days from symptom onset		**≤11**		**11**		**7**	7		**13**	
Hypertension (%)	38	46	48	37	40	45	21		52	34
Type-2 Diabetes (%)	9.5	14		12	20	13	11			6
Heart disease (%)	9.5	14		18	13	12	8			
Glucocorticoids (%) (Methylprednisolone dose)	100 **(MP 1 mg/Kg)**	0	**38** **(MP 1 mg/Kg)**	**0**	0	30	**0**		**48** **(MP 2-3 mg/Kg)**	**100** **(MP 0.5-1 mg/kg)**
Remdesivir (%)			≤7					9.5	0	0
Ambient air (%)				0	0		0		0	0
LFO (%)				0	0		0		8	4
HFO or NIV (%)				100	83		100		72	96
IMV (%)			**62**	0	**17**	**18**	0		**20**	**0**
(Median) PaO2/FIO2 ratio (mmHg)	**225**	**(≤300)**		**107**	**109**	**169**	**< 100 in 60%**	**180**	(mean) **104**	
(Median) CRP (mg/L)	**21**	**123**	**79**	**156**	**130**	34-61 (partial data)	143		(mean) **198**	
(Median) Ferritin (ng/mL)				**1400**		**1168 (partial data)**	**1849**		(mean) **1638**	(mean) **1592**
(Median) D-dimer (ng/mL)			835			**1000-1210 (partial data)**	1270		(mean) **3801**	(mean) **2931**
(Median) LDH (U/L)	445		**625**	**469**	**505.5**	600-676 (partial data)	468		(mean) **524**	
**OUTCOMES (treatment *vs.* standard-of-care):**										
**(28-day) Clinical status Improvement (%)**				**69 vs. 61** **(p=NS)**			**17 vs. 18**			**(7-day CPAP)** **8 vs. 87**
**(28-day) Discharge (%)**		**92 vs. 42**	**(35-day)** **24 vs. 100**	**63 vs. 49** **(p=NS)**			**17 vs. 17**			**(overall)** **88 vs. 64.5**
(**28-day) IMV (%)** **[HR]**	**(7-day ICU)** **14 vs. 13**			**13 vs. 6** **(p=NS)**	**[HR 0.615, p=0.072]**	(14-day) 18 vs. 16	**6 vs. 7**		**(30-day)** **17 vs. 48 (p=0.001)** **[HR 0.36, p=0.017]**	
**(28-day) IMV or death (%) [HR]**						**[Adj HR 0.61, p=0.020]** **(For P/F ≤ 150:** **Adj HR 0.19, p=0.011; significance kept for IV administration only:** **Adj HR 0.55, p=0.042)**		35 vs.?		
**(28-day) Mortality (%)** **[HR]**	**(7-day)** **24 vs. 21** **[OR 0.78,** **p=NS]**	**8 vs. 58** **[HR 0.035, p=0.004]**	**(35-day)** **9.5 vs. 0**	**16 vs. 33** **(p=NS)**	**(30-day)** **33 vs. 53** **[HR 0.46, p=0.040]**	**(14-day)** **7 vs. 20 (p=0.0007)** **[Adj HR 0.38, p=0.015]** **(Significance kept for IV administration only:** **Adj HR 0.29, p=0.048)**	**2 vs. 5** **(p=NS)**	**[HR 0.50, p=0.035]**	**(30-day)** **27 vs. 38** **[HR 0.61,** **p=NS]**	**(overall)** **12 vs. 34**
Infections (%)		0 vs. 0	65 vs. 0	13 vs. 12 **(6% one dose vs. 33% two doses,** **p=0.06)**	43 vs.?	**13 vs. 4** **(p<0.0001)**	21 vs. 18	32 vs.?	31 vs. 39	0

LEGEND OF COLOURS: Green: numbers falling within the thresholds herein suggested (optimal study and patient characteristics), in relation with statistically significant results and substantial benefit on overall mortality (i.e., positive outcomes). Blue: numbers falling borderline (suboptimal study and patient characteristics), in relation with numerically but not statistically significant results, and substantial benefit on clinical status (e.g., IMV requirement, hospital discharge) (i.e., partially positive outcomes). Red: numbers falling outside of the thresholds (non-optimal study and patient characteristics), in relation with non-significant results, and no substantial benefit on either clinical status or overall mortality (i.e., negative outcomes).

Adj, adjusted; HR, hazard ratio; NS, not significant.

It was noteworthy that most of the studies with positive results had recruited at least 50 patients in the treatment arm (46, 50, 52, 54), whereas those showing negative or non-significant results included fewer patients (45, 47, 48, 51), thus suggesting that results might have been influenced by the small size of the cohorts. Most of the patients were men (≥ 70%), aged between 59 and 64 years old; however, some studies with negative or partial results included more women (53) and/or patients that were on average older than 70 (53) or younger than 59 (51). No obvious difference in the overall distribution of comorbidities (e.g., hypertension, diabetes, heart disease) was noticed between studies with positive and negative results.

According to the data available, patients were generally enrolled for treatment within the second week of symptom onset and within 4 days of hospitalisation. Tocilizumab was given mainly intravenously, at various doses (400-800 mg), in single or multiple infusions (1 to 3 infusions, at intervals of 12-72 hours). It was noted that two infusions could increase the rate of secondary infections and bacteremia compared to a single infusion (48, 50), while the effects of subcutaneous administration on mortality and composite outcomes (i.e., IMV + death) lost statistical significance in the subgroup analysis (50).

Concerning concomitant therapies, standard of care originally included drugs (e.g., hydroxychloroquine, azithromycin, lopinavir/ritonavir) subsequently proved ineffective against COVID-19 (55, 56), while remdesivir was not available or administered to a negligible number of patients (47, 52). Since the RECOVERY data on the efficacy of intermediate-to-low doses of dexamethasone and the benefit of combining tocilizumab with dexamethasone on overall survival were published later (57, 58), standard of care and treatment protocols did not yet include the use of glucocorticoids. In some cases, steroids were given to a minority of patients (<50%) (47, 50, 53), generally at high doses (i.e., methylprednisolone 1-2 mg/Kg per day) as supportive therapy (45, 53). In one study, in which all tocilizumab patients were co-treated with lower doses of steroids (i.e., methylprednisolone 0.5-1 mg/Kg per day, tapered and suspended within 7-10 days), tocilizumab showed good results (54).

Most importantly, patients who successfully responded to IL-6 inhibitors had severe disease, but neither critical nor moderate disease. Studies in which most of the patients required IMV showed negative results (47), whereas treatment was effective in case series without or with low numbers of intubated patients (49, 50, 54). On the other hand, due to the overwhelming of ICU during the first months of the outbreak, patients that underwent IMV were probably less than they should have been (48, 51). Parameters to be considered more reliable of disease severity could instead be the pressure of arterial oxygen to fractional inspired oxygen concentration (PaO2/FIO2) ratio and possibly some inflammatory markers. In fact, patients from studies reporting significantly reduced mortality with IL-6 inhibitors had baseline median PaO2/FIO2 ratios between 110 and 200 mmHg (i.e., severe disease) (46, 49, 50, 52); studies with median PaO2/FIO2 ratios between 100 and 110 mmHg (i.e., severe-to-critical disease) showed numerically but not statistically significant differences in mortality (48, 53) and in some cases reported a significant reduction in IMV requirement (53); whereas studies with median PaO2/FIO2 ratios < 100 (i.e., critical disease) or > 200 mmHg (i.e., moderate disease) failed to show substantial differences in mortality, IMV requirement or hospital discharge (45, 47, 51).

Looking at inflammatory markers, studies with positive results reported median CRP values around 120-130 mg/L (46, 49), whereas studies with only partial results or negative results reported values ≥ 160 mg/L (48, 53) or ≤ 80 mg/L (45, 47). Moreover, patient series with ferritin levels < 1600 ng/mL and D-dimer levels < 3000 ng/mL (50, 54) showed better outcomes compared to those with higher values (51, 53) (**Table 1**).

Univariate and multivariate analyses confirmed many of these observations. Compared to patients who did not respond to IL-6 inhibitors, patients who improved after treatment showed significant differences in baseline median values of: age (59-63 versus 74 years old) (48, 59), male percentage (100 versus 70%) (48), PaO2/FIO2 ratio (112-137 versus 81-86 mmHg) (48, 51), CRP (128 versus 186 mg/L) (48), IL-6 (52-58 versus 99-120 pg/ml) (51, 59), and neutrophil-to-lymphocyte ratio (3.5 versus 8) (59). Similar numerical differences in age, PaO2/FIO2 ratio, IL-6 and neutrophil-to-lymphocyte ratio were actually observed between ICU patients requiring IMV and non-ICU patients requiring NIV/HFO (47, 59), further indicating that IL-6 blockade responders belong to this second category of patients.

At multivariate regression analysis, independent predictors of clinical improvement and survival upon treatment were: age ≤ 70-75 years old (48, 59, 60), PaO2/FIO2 ratio ≥ 100 (48, 51, 60), IL-6 values < 91 pg/mL (59), administration of tocilizumab within 6 days (61), and no need of IMV (60). Conversely, independent predictors of ICU admission and mortality were: comorbidities (in particular kidney impairment) (53), IMV at baseline (9, 53), and D-dimer levels > 3500 ng/ml (61). Both tocilizumab and glucocorticoid use were predictors of reduced mortality at 14 days, in the case of tocilizumab specifically in patients not requiring IMV (62).

After treatment, good responders showed control values of ferritin returning < 1400 ng/ml, D-dimer < 3000 ng/ml and lactate dehydrogenase (LDH) < 400 U/L, while non-responders had ferritin > 1800 ng/ml, D-dimer > 5000 ng/ml and LDH > 500 U/L (63). Lower levels of IL-6 24 hours after the infusion were found predictive of clinical improvement at day 7 (62). Irrespective of the main outcome, IL-6 inhibitors led to a fast decrease and normalisation of CRP levels, and a rapid resolution of fever and fatigue (45, 50, 51, 54, 63).

## More Conclusive Evidence: Results From Ramdomised Controlled Trials

From the early stages of the pandemic, IL-6 has been a well-documented biomarker of disease severity and poor prognosis, and considered among the most promising targets in the treatment of the COVID-19 cytokine storm (19). About half of the clinical trials being conducted worldwide testing monoclonal antibodies for the management of severe COVID-19 have been performed with IL-6 inhibitors, and in particular with tocilizumab (64).

As of May 1, 2021, 11 RCTs with tocilizumab (10 RCTs) and/or sarilumab (2 RCTs) were published in peer-reviewed journals (43, 58, 65–73). Many of the observations made in the above reported Italian observational studies were confirmed in the RCTs. Studies were heterogenous in sample size, patient composition (e.g., age, sex, ethnicity), treatment protocol (e.g., dosage, number and timing of infusions), concomitant therapies (e.g., glucocorticoids, antivirals), and disease severity (e.g., respiratory status, oxygen requirement, inflammatory markers) (**Table 2**).

**Table 2:** Randomised controlled trials with IL-6 inhibitors in COVID-19.

PROVED EFFICACY	YES	YES	PARTIAL	NO	NO (YESin “severe subgroup”)	NO	NO	NO (PARTIAL)	NO	PARTIAL	NO
**TRIAL CHARACTERISTICS:**											
Authors [Ref.]	Horby et al. (58)	Gordon et al. (65)	Rosas et al. (66)	Wang et al. (67)	Soin et al. (68)	Lescure et al. (69)	Veiga et al. (43)	Salama et al. (70)	Stone et al. (71)	Hermine et al. (72)	Salvarani et al. (73)
Trial name (Geographic area)	RECOVERY (UK)	REMAP-CAP (Multinational)	COVACTA (Multinational)	(China)	COVINTOC (India)	(Multinational)	TOCIBRAS (Brazil)	EMPACTA (Multinational)	BACC Bay (USA)	CORIMUNO-TOCI 1 (France)	RCT-TCZ- COVID-19 (Italy)
Date of (online) publication (preprints or updates)	May 1, 2021 (preprint Feb 11, 2021)	Apr 22, 2021 (preprint Jan 9, 2021)	Apr 22, 2021 (preprint Sep 12, 2020)	Mar 9, 2021	March 4, 2021	March 4, 2021 (preprint Feb 3, 2021)	Jan 20, 2021	Dec 17, 2020	Oct 21, 2020 (updated Nov 19, 2020)	Oct 20, 2020 (corrected Jan 4, 2021)	Oct 20, 2020
Period of recruitment	Apr 23, 2020 - Jan 24, 2021	Mar 9 – Nov 19, 2020	Apr 3 – May 28, 2020	?	May 30 – Aug 31, 2020	Mar 28 – Jul 3, 2020	May 8 – Jul 17, 2020	? – Sep 30, 2020	Apr 20 – Jun 15, 2020	Mar 31 – Apr 18, 2020	Mar 31 – Jun 11, 2020
Design of the study	Open-label	Open-label	Double-blind	Open-label	Open-label	Double-blind	Open-label	Double-blind	Double-blind	Open-label	Open-label
Modified intention-to-treat population	4116	803	438	55	180	416	129	377	242	131	123
N. Patients in the treatment arm	**2022**	**401** [**353** (toci) **plus** **48** (sari)]	**294**	**33**	**91**	**159** (200 mg arm) **and** **174** (400 mg arm)	**65**	**249**	**161**	**64**	**60**
Treatment arm (route, dosage, number of doses, intervals)	Tocilizumab IV 400-800 mg (≈ 8mg/Kg) (1 + 1 optional after 12-24h)	Tocilizumab IV 8 mg/kg (max 800 mg) (1 + 1 optional after 12-24h); or Sarilumab IV 400 mg (single dose)	Tocilizumab IV 6 mg/Kg (max 480 mg) (1 + 1 optional after 8-24h)	Tocilizumab IV 400 mg (1 + 1 optional after 24h)	Tocilizumab IV 6 mg/Kg (max 480 mg) (1 + 1 optional after 12-168h)	Sarilumab IV 200-400 mg (single dose)	Tocilizumab IV 8 mg/Kg (single dose)	Tocilizumab IV 8 mg/Kg (1 + 1 optional after 8-24h)	Tocilizumab IV 8 mg/Kg (max 800 mg) (single dose)	Tocilizumab IV 8 mg/Kg (1 + 1 optional after 48h)	Tocilizumab IV 8 mg/Kg (max 800 mg) + second dose after 12h
**PATIENT CHARACTERISTICS (treatment arm):**											
(Median) Age (years old)	**63**	(mean) **61.5-63**	(mean) **61**	63.5	**56**	**58-58**	(mean) **57**	(mean) **56**	62	**64**	61.5
Male sex (%)	66	**74-81**	**70**	**53**	**84**	**68-57**	(mean) **68**	**60**	**60**	**70**	**67**
Race/Ethnicity (%)	**White 76**, Black+Asian+Other 18, Unknown 7	**White 70-74**, Asian 18-21, Black 5-3, Other 7-3	**White 60**, Black 14, Asian 9.5, Other 3.5, Unknown 13	**Asian 100**	**Asian 100**	White 79-74 (**Hispanic 33-38**), Black 2-3, Asian 3-5, Other 16-18	**Hispanic 100**	White 68.5 (**Hispanic 57.5**), Black 14, Other 13.5, Unknown 4	**White 44**, Black 15, Asian 4, Other 23, Unknown 14		
(Median) BMI		30.5-29			(mean) 27			(mean) 32	30	28	
Obesity (%)			21			23-21	23		**50**		28
Type-2 Diabetes (%)	28		36	12	34	28-27	34		28	33	17
Hypertension (%)			**60.5**	29	**40**	**43-40**	**46**		**50**		**45**
Cardiovascular impairment (%)	22		30		16	4-5	12		20	33	
Chronic Lung Disease (%)	23		17		5	9-7	9		18	13	3
(Severe) Hepatic impairment (%)	1		2								
(Severe) Kidney impairment (%)	**1**				4	4-3	**8**		**18**	**8**	
N. Days from hospitalization	**2**	**1-1** **(1 day - median 14 hours - from ICU admission)**	3			3-2 **(2 days from ICU admission)**				1	2
N. Days from symptom onset	9		12	**20**		5-4	10		9	10	7
Glucocorticoids (%)	**82**	**87-91**	**34**	**15**	**91 (96 in “severe”)**	**16-24** (varying during the study)	**69**	**80**	**11**	**33**	**0**
Remdesivir (%)		33			**43 (42 in “severe”)**	**<1**	**0**	**53**	33		**0**
(Median) PaO2/FIO2 ratio (mmHg)		**115-126**				**(SpO2/FiO2 ratio) 230-237.5**					**262.5**
Ambient air (%)	0.5	<1-0	3	23	11 (2 in “severe”)	1-0	0	9	14	0	0
LFO (%)	**45.5**	**<1-0**	**27**	**68**	**53 (42 in “severe”)**	**70-74**	**60**	**65**	**83**	**100**	**100**
HFO or NIV (%)	**41**	**71-83**	**32**	**9**	**31 (46 in “severe”)**	**18-12**	**23**	**26**	**3**	**0**	**0**
IMV or ECMO (%)	**13**	**29-17**	**38**	0	**5 (10 in “severe”)**	11-14	17	0	0	0	0
(Median) CRP (mg/l)	**143**	**150-136**	**157**		**(mean) 111** (N/A in “severe”)	**94-96**	**(mean) 160**	**124.5**	**116**	**119.5**	**105**
(Median) Ferritin (ng/ml)	**947**		**(mean) 3066.5**		**(mean) 921** (N/A in “severe”)	**695-737**	(mean) 1271	**624**	**723**	**1292**	**646**
(Median) D-dimer (ng/ml)		**832-828**				**480-540**	1000	**1600**	857	**869**	756
**OUTCOMES (treatment *vs.* standard-of-care):**											
Clinical status or functional Improvement (%)		**21-day median organ support-free days:** **10-11 *vs.* 0** **(Adjusted OR** **1.64-1.76, probability of superiority >99%)**	**28-day median ordinal scale:** **1 *vs.* 2 (p=NS);** **Median time to improvement:** **14 *vs.* 18 days (HR 1.26)**	**14-day, “cure rate”: 94 *vs.* 87 (p=NS);** **14-day hypoxia recovery rate:** **92 *vs.* 60, p=0.033)**		**Median time to** **≥ 2-point improvement:** **10-10 *vs.* 12 days** **(HR 1.03-1.14, p=NS)**		**Median time to improvement:** **6 *vs.* 7 days** **(HR 1.15)**	**91 *vs.* 89** **(HR 1.08);** **Weaned from oxygen:** **83 *vs.* 85**	**Weaned from oxygen:** **89 *vs.* 75**	
(28-day) Discharge (%)	**57 *vs.* 50 (p<0.0001) (RR 1.22);** **Median time to discharge: 19 *vs.* >28 days)**		**Median time to discharge:** **20 *vs.* 28 days (HR 1.35, nominal p=0.04 in preprint** **version)**	**Time to discharge: 26 *vs.* 24 days**				**Median time to discharge:** **6 *vs.* 7.5 days** **(HR 1.16)**	**91 *vs.* 89** **(HR 1.08)**	**83 *vs.* 73**	**(30-day)** **90 *vs.* 92** **(RR 0.98)**
(28-day) Progression (%)			**ICU transfer:** **21 *vs.* 36**		**12 *vs.* 18 (p=NS);** **16 *vs.* 34 (p=0.044) in “severe”**				**19 *vs.* 17** **(HR 1.11, p=NS)**	**(14-day) ventilator-free survival:** **24 *vs.* 36** **(HR 0.58, probability of HR<1 = 95%)**	**(30-day)** **ICU transfer:** **10 *vs.* 8** **(RR 1.26)**
(28-day) IMV (%)	**15 *vs.* 19** **(RR 0.79, p=0.0019)**		**28 *vs.* 37**		**15 *vs.* 15 (p=NS)**						
**(28-day) IMV + death (%)**	**35 *vs.* 42** **(RR 0.84, p<0.0001)**		**ICU+IMV+death** **(clinical failure): 29 *vs.* 42** **(HR 0.61, nominal p=0.03 in preprint version)**				**(15-day)** **28 *vs.* 20** **(OR 1.54, p=NS)**	**12 *vs.* 19** **(HR 0.56, p=0.04)**	**11 *vs.* 12.5** **(HR 0.83, p=NS)**	**(14-day)** **17 *vs.* 27** **(HR 0.58, probability of HR<1 = 92.5%)**	
**(28-day) Mortality (%)**	**31 *vs.* 35** **(HR 0.85, p=0.0028);** **(steroids *vs.* no steroids: death RR** **0.79 *vs.* 1.16, interaction p=0.01)**	**(21-day)** **28-22 *vs.* 36** **(adjusted OR 1.64-2.01, probability of superiority** **>99%)**	**20 *vs.* 19** **(p=NS)**	**0 *vs.* 0**	**12 *vs.* 17 (p=NS);** **16 vs 34 in “severe”** **(p=0.044)**	**(29-day)** **10-8 *vs.* 8** **(p=NS)**	**21 *vs.* 9** **(OR 2.70, p=0.07)**	**10 *vs.* 9** **(p=NS)**	**6 *vs.* 4** **(HR 1.52)**	**11 *vs.* 12** **(HR 0.92, p=NS)**	**(30-day)** **3 *vs.* 1.5** **(RR 2.10,** **p=NS)**
Serious adverse events (%)	3 *vs.* 0	9-0 *vs.* 11	35 *vs.* 38.5	0 *vs.* 3	20 *vs.* 17	26-29 *vs.* 24	16 *vs.* 11 (p=NS)	15 *vs.* 20		32 *vs.* 43 (p=NS)	1 *vs.* 2 events
Serious infections (%)	3 *vs.* 0	1-0 *vs.* 0	21 *vs.* 26		3 *vs.* 0	11-13 *vs.* 12		5 *vs.* 7	**8 *vs.* 17** **(p=0.03)**	**3 *vs.* 16**	0 *vs.* 2 events

LEGEND OF COLOURS: Green: numbers falling within the thresholds herein suggested (optimal trial and patient characteristics), in relation with statistically significant results, and significant benefit on overall mortality (positive outcomes). Blue: numbers falling borderline (suboptimal trial and patient characteristics), in relation with numerically (i.e., >20% differences) but not statistically significant results, and significant benefit on composite endpoints (IMV+death) but not on overall mortality (partially positive outcomes). Red: numbers falling outside of the thresholds (non-optimal trial and patient characteristics), in relation with non-significant results, and no benefit on either composite endpoints or overall mortality (negative outcomes).

HR, hazard ratio; OR, odds ratio; RR, relative risk; NS, not significant.

Many studies with negative results recruited fewer than 100 patients into the treatment arm (43, 67, 68, 73), while the best outcomes were reported in the two largest trials (i.e., RECOVERY and REMAP-CAP) (58, 65), again suggesting that some inconclusive results may have suffered from low statistical power. Studies with worse results also included patients younger (< 59 years old) (43, 68–70) than other studies with better results (61-64 years old) (58, 65, 66, 72). Excluding RECOVERY, studies with overall negative outcomes included fewer men (< 70%) (43, 67, 69–71, 73) compared to other studies with positive or partially positive outcomes (≥ 70%) (65, 66, 72). Whereas successful trials included high numbers of Caucasian White patients (≥ 70%) (58, 65), worse results were obtained in trials with fewer White patients and relatively more Hispanic White and/or Black Afroamerican patients (43, 69–71), or with Asian patients (67, 68). Studies with poor outcomes also included numerous patients with comorbidities (e.g., obesity, hypertension, kidney impairment) (43, 68, 69, 71).

Treatment was given intravenously, at various doses (tocilizumab 400-800 mg or 6-8 mg/Kg, in 1-2 infusions, at variable intervals ranging from 8 hours to 7 days; or sarilumab 200-400 mg in a single infusion), and was mostly started within the first 3 days of hospitalisation and/or within 2 weeks from symptom onset. Importantly, in severe-to-critical patients, treatment was successful if given within 24 hours from ICU admission (65), but not later (69).

In successful trials, standard of care included glucocorticoids, which were then administered to the great majority of the patients (> 80%) (58, 65), whereas substantially fewer patients were co-treated with steroids in studies with negative results (43, 67, 69, 71, 73). Antivirals, and in particular remdesivir, were given in variable proportion, without obvious implications in the overall outcome.

Most remarkably, best responses on disease progression and mortality were obtained in trials enrolling a substantial proportion of patients requiring HFO or NIV at baseline (> 40%, ie. severe disease), a limited amount of patients requiring LFO (< 50%, ie. moderate disease), and only a minority of patients requiring IMV (< 30%, ie. critical disease) (58, 65). Even in COVINTOC trial (68), an Indian RCT reporting overall negative or partial results, positive outcomes were observed when analysing specifically the “severe subgroup”, in which these proportions were respected. As observed for earlier Italian studies (although the data here are largely lacking), a baseline median PaO2/FIO2 (or SaO2/FIO2) ratio > 110 mmHg was reported in a successful trial (65), whereas values > 200 mmHg were reported in negative trials (69, 73).

Concerning acute-phase reactants, in line with previous Italian cohort studies, trials with positive outcomes reported baseline median CRP values overall ranging between 120 and 155 mg/L. In particular: good efficacy (i.e., significantly reduced progression and mortality) was reported in trials with CRP values between 130 and 150 mg/L (58, 65); partial efficacy (i.e., significantly reduced progression including ICU transfer and IMV rate, but not overall mortality) was observed in trials with CRP values between 120 and 125 mg/L (70, 72) or around 155 mg/L (66); and no efficacy was obtained in trials with CRP values ≤ 115 mg/L (69, 71, 73) or ≥ 160 mg/L (43). Furthermore, whereas the two major successful trials largely included patients with only modestly increased levels of ferritin and D-dimer (58, 65), trials that reported poor outcomes mostly included patients with lower (69, 71, 73) or higher (43) values of ferritin (< 800 or > 1200-1400 ng/ml) and D-dimer (< 700 or > 1000-1500 ng/ml), and trials that reported partial efficacy showed dichotomous trends (70, 72).

No significant increase in serious adverse events associated with IL-6 inhibitors was noted. Indeed, in some trials, serious infections were numerically lower in the treatment arm (66, 71, 72), and significantly lower in one study (71) (**Table 2**). Results from the MARIPOSA study suggest a more favourable profile for tocilizumab at a dose of 8 mg/Kg versus 4 mg/Kg in terms of lower mortality and fewer serious adverse events (74).

## Confirmations From Meta-Analyses and Current Guidelines

Early meta-analyses of the first published RCTs did not show significant differences in mortality with anti-IL-6R agents, although they showed significantly lower rates of ICU transfer, IMV, and composite outcome of IMV or death, along with a lower rate of serious infections (22, 75, 76). This could have been due to the small size and composite primary endpoints of early trials, probably underpowered to detect differences in mortality, and to the high heterogeneity among the studies. However, subsequent meta-analyses of more recently completed RCTs including RECOVERY, or even unpublished data through searches of electronic databases and contact with experts, ultimately concluded that, in patients hospitalised with COVID-19, treatment with IL-6 antagonists, and in particular tocilizumab (with much higher certainty than sarilumab, but not siltuximab), results into a significant reduction in 28-day all-cause mortality (2, 32, 58), in line with previous meta-analyses of observational cohort studies (22, 77, 78). Specifically, high probability of reduced risk of mortality and clinically meaningful mortality benefit was observed in patients receiving concomitant glucocorticoids and non-invasive ventilation or high-flow oxygen, with no need of IMV or cardiovascular support (32, 79). A significant interaction was seen in the subgroup analysis in regard to concomitant steroid therapy (2), while other meta-analyses confirmed the independent strong benefit of steroid therapy, particularly low-dose dexametasone (not high-dose methylprednisolone or hydrocortisone), on survival of severe and critical patients (80). Lower odds of mortality with tocilizumab were observed in patients with CRP values between 75 and 150 mg/L compared to patients with lower or higher levels; yet, a statistical significance was not reached (32).

According to the NIH COVID-19 treatment guidelines (81), largely based on the findings and recruiting criteria of RECOVERY and REMAP-CAP trials, tocilizumab (at a single intravenous dose of 8 mg/Kg actual body weight up to 800 mg) or sarilumab (in case tocilizumab is not available, at a single intravenous dose of 400 mg) are recommended, in combination with dexamethasone (6 mg per day, intravenously, for up to 10 days), for recently hospitalised patients (within 3 days from hospital admission and/or within 24 hours from ICU admission), with a rapid respiratory decompensation requiring increasing amounts of oxygen (in particular, HFO or NIV, or soon after starting IMV), and substantially increased markers of inflammation (e.g., CRP ≥ 75 mg/L).

## Therapeutic Windows: To Cure the Right Patient at the Right Time We Need Clear Cut-Offs

A correct stratification of the patients is crucial for achieving satisfactory results with tocilizumab in the treatment of COVID-19, in clinical trials as in clinical practise. Although only large individual patient data meta-analyses would best indicate which patients are more likely to benefit from IL-6R blockade, from this review of the data we aimed to identify, for some important respiratory and inflammatory parameters, the minimum and maximum thresholds between which to build up a therapeutic window.

Relatively simple numerical cut-offs would actually reflect the complexity and heterogeneity of disease biology, helping to recognize the stage and progression of the disease, the prevailing pathogenic aspects, the predominant inflammatory patterns, and the actual significance of IL-6 in a given patient.

IL-6 levels are significantly correlated with worsening oxygen exchange in the lungs of COVID-19 patients and are major predictors of disease progression and mortality (23). Plasma levels of IL-6 < 7-10 pg/ml are found in healthy subjects (and presumably asymptomatic carriers); < 20 pg/ml (median 17 pg/ml) in low-risk patients (i.e., mild-moderate disease); > 35 pg/ml (around 2/3-fold higher median values) in patients at risk of progression (i.e., moderate-to-severe disease); > 55 pg/ml in patients with a complicated course (i.e., severe disease); and > 80 pg/ml (median values 86-91 pg/ml) in patients developing severe respiratory failure requiring ventilatory support and at high risk of in-hospital death (i.e., severe-to-critical and critical disease) (82–87). These thresholds are in line with results of univariate and multivariate analysis of the Italian observational studies exposed above (51, 59). Interestingly, daily monitoring of IL-6 levels showed that, in severe progressive forms, IL-6 increases up to 85-90 pg/ml at 6-7 days of hospitalisation, and reaching these values precedes a further increase to peak concentrations above 120-300 pg/ml on days 9-10 (88). Acting within 6 days of hospitalisation in severe patients, identified by means of baseline IL-6 values between 35 and 90 pg/ml, may therefore represent the right time to interrupt the inflammatory cascade and prevent lethal cytokine storm.

A good response to IL-6R blockade is expected when IL-6 elevation is accompanied at baseline by an increase in CRP values. Higher levels of both IL-6 and CRP are, in fact, associated with disease severity in COVID-19 (89). Indeed, cytokine blockade was not effective in COVID-19-unrelated ARDS (showing higher IL-6 levels but much lower CRP values as compared to severe COVID-19), while it is effective in CAR T-cell induced CRS (showing high concentrations of both IL-6 and CRP) (90). On the other hand, tocilizumab does not reduce serum IL-6 concentrations, which are instead variably increased upon treatment, but it does efficiently reduce CRP levels, which reflect IL-6 bioactivity. CRP levels < 80-100 mg/L (median 77 mg/L) are found in patients at low risk of progression (i.e., mild-moderate disease), whereas CRP levels > 180-200 mg/L (median 194 mg/L) are detected in patients with high rates of mortality (i.e., critical disease) (86, 87). From the observational studies and RCTs reported above, it emerges that patients with CRP values between 120 and 160 mg/L are actually those with a severe progressive disease who are most likely to respond successfully to IL-6 inhibitors.

In regard to serum ferritin levels, observations from the aforementioned retrospective cohort studies and RCTs seem to confirm other reports (91, 92), according to which low-risk patients can be identified for values < 400-800 ng/ml (i.e., mild-moderate disease); patients at high risk of progression for values between 800 and 1400-1600 ng/ml (i.e., severe progressive disease); and patients at high risk of death for values > 1600-2000 ng/ml (i.e., critical disease). Severe hyperferritinemia (> 1600-2000 ng/ml) is a criterion for MAS (93, 94), and probably indicates an overt state of MAS-like hyperinflammation in COVID-19 that specifically occurs in critically-ill patients (19). Due to the postulated regulatory role of IL-6 at this stage, tocilizumab would be no longer effective in these cases; conversely, activation of the NLR family pyrin domain containing 3 (NLRP3) inflammasome, which is typical of hyperferritinemic syndromes, would be crucial also in critical COVID-19 (7), and therapies targeting IL-1, such as anakinra, could still be effective (95). In addition to being associated with intense macrophage activation and release of IL-1, high ferritin levels are also associated with severe hepatocellular damage and LDH elevation, as well as with development of diffuse microthrombosis and coagulopathy with severely increased D-dimer concentrations (19). In fact, high D-dimer levels were found to predict mortality and poor response to tocilizumab in COVID-19 patients (61, 96, 97); indeed, treatment with tocilizumab could even lead to a further increase in D-dimer, despite the decrease in fibrinogen (52, 63). Altogether, COVID-19 patients characterised by serum ferritin levels > 1600-2000 ng/mL, LDH > 500-550 U/L, and D-dimer > 3000-5000 ng/mL, are most likely hyperinflamed and critically-ill patients who do not respond to IL-6R blockade (but may still respond to IL-1 blockade). It remains to be assessed whether, in addition to these absolute thresholds, the ratio of CRP to ferritin (or D-dimer) may inform the advisability of using tocilizumab or anakinra (or full-dose anticoagulants). Most recently, the soluble urokinase plasminogen activator receptor (suPAR) has been proposed as an alternative and more sensitive biomarker for early identification of those COVID-19 patients at risk of progression who release high amounts of alarmins S100A8/A9 and IL-1α and particularly respond to anakinra, thus allowing the extension of the use of anakinra even to more moderate forms of disease (98).

Concerning respiratory parameters and oxygen requirements, as exposed in the previous paragraphs, RCTs and meta-analyses have concluded that the best responders to IL-6 inhibitors, particularly tocilizumab, are hypoxic and severely progressing patients requiring high-flow oxygen or non-invasive ventilation, while from retrospective cohort studies it can be suggested that this subgroup basically corresponds to patients with values of PaO2/FIO2 ratio between 100 and 200 mmHg. In this subset of patients, median values of CRP, ferritin, D-dimer, LDH and neutrophil-to-lymphocyte ratio in blood actually fall within the optimal ranges proposed here (47, 59, 95). By contrast, critically-ill patients (showing values of PaO2/FIO2 ratio <100 mmHg and requiring IMV) and mild-moderate patients (showing values of PaO2/FIO2 ratio > 200-300 mmHg and requiring low-flow or no oxygen) generally show poor responses to IL-6 inhibitors. Nevertheless, results from a large RCT evaluating sarilumab recently posted as a preprint (99) seem to suggest that IL-6 inhibitors can reduce the risk of death by up to half even in ICU patients undergoing IMV, in case these are co-treated with steroids and still show relatively low median values of IL-6 and CRP, substantially falling within our proposed response ranges.

**Figure 3:**
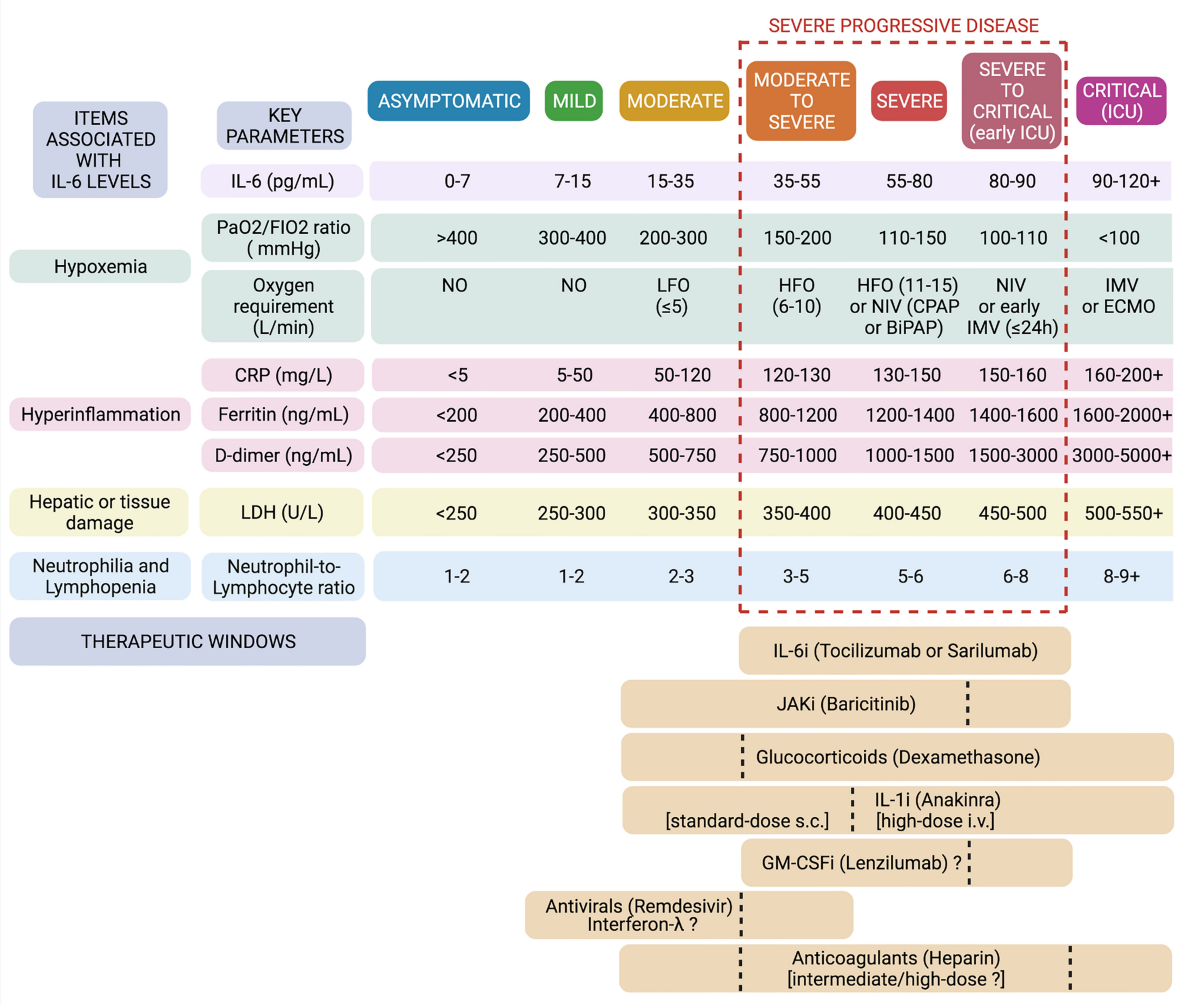
Defining therapeutic windows for IL-6 inhibitors and other biologics in COVID-19. In the light of baseline characteristics of previous successful and unsuccessful trials, univariate and multivariate analyses, meta-analyses, and studies on the predictivity of IL-6 and other biomarkers, we set thresholds and ranges for some major respiratory and laboratory parameters in the attempt to frame specific windows of opportunity for tocilizumab and other agents in the immunotherapy of COVID-19. Created with Biorender.com.

A schematic summary of the suggested items and cut-offs to consider in COVID-19 to discern good and bad responders to tocilizumab (and other agents) is provided in **Figure 3**.

## Going Beyond IL-6

In managing COVID-19, growing interest is turning to Janus kinase (JAK) inhibitors. Among these, baricitinib was originally identified using artificial intelligence as a suitable candidate for COVID-19 treatment, due to its ability to bind with high affinity to some key regulators of clathrin-mediated endocytosis, namely AP2-associated protein kinase-1 (AAK1) and possibly cyclic G-associated kinase (GAK), thus inhibiting the entry of SARS-CoV-2 into cells and the intracellular assembly of virus particles (100). Such antiviral effects were more recently demonstrated *in vitro* using 3D cultures of primary human liver cells, in which baricitinib was able to reduce SARS-CoV-2 infectivity and viral load by directly inhibiting numb-associated kinases and impeding IFNα2-mediated induction of the SARS-CoV-2 receptor angiotensin-converting enzyme 2 (ACE2) (101). Plasma concentrations of baricitinib at therapeutic doses would be sufficient to exert antiviral effects *in vivo* (100).

Moreover, and perhaps most importantly, JAK inhibitors exert multidirectional anti-inflammatory effects by simultaneously inhibiting intracellular signals induced by several cytokines. Acting on JAK1 would broadly interfere with the signalling downstream of cytokine receptors belonging to the gp130 family (e.g., IL-6, IL-27), IFN family (e.g., IFNγ), γ-chain family (e.g., IL-4, IL-9, IL-7) and others (e.g., thymic stromal lymphopoietin, IL-13, G-CSF); acting on JAK2 would specifically interfere with GM-CSF, IL-23, IL-12 and IL-6 signals; while acting on JAK3 would more potently interfere with the γ-chain receptor family (102).

Different JAK inhibitors have been proposed for the treatment of COVID-19, namely: JAK1/JAK2/TYK2 inhibitors (e.g., baricitinib, ruxolitinib) (103–109), selective JAK2 inhibitors (e.g., fedratinib) (110), and - more recently - JAK1/JAK3 inhibitors (e.g., tofacitinib) (111). Probably due to their ability to block various inflammatory pathways including not only IL-6/STAT3 and TH17 (advanced stages), but also TH9 and TH1/TH-GM pathways (upstream stages) (**Figure 2**) (7, 104–106), in addition to their presumed direct effects on viral infection and replication (early stages) (100, 101), JAK inhibitors appeared from both observational studies (103, 104, 106) and RCTs (107–109, 111) to be effective not only in severe forms of COVID-19, but also in patients with moderate disease (**Figure 3**), the latters representing the vast majority of patients in these studies (**Table 3**). Furthermore, baricitinib may work even in the absence of concomitant steroids, and in series involving more women and younger patients (103, 104, 108, 109). Nevertheless, as with tocilizumab, the most significant results with baricitinib (on time-to-recovery or all-cause mortality, according to ACTT-2 and COV-BARRIER trials, respectively) were obtained in patients with severe disease requiring at baseline HFO or NIV (81, 108, 109) (**Table 3**).

**Table 3:** Randomised controlled trials with JAK inhibitors in COVID-19.

PROVED EFFICACY	PARTIAL	YES	YES	YES
**TRIAL CHARACTERISTICS:**				
Authors [Ref.]	Cao et al. (107)	Kalil et al. (108)	Marconi et al. (109)	Guimaraes et al. (111)
Study name (Geographic area)	(China)	ACTT-2 (Multinational)	COV-BARRIER (Multinational)	STOP-COVID (Brazil)
Design of the study	Single-blind	Double-blind	Double-blind	Double-blind
Date of (online) publication	May 26, 2020	Dec 11, 2020	Sep 1, 2021 (Preprint posted May 30, 2021)	Jun 16, 2021
Period of enrollment	Feb 9 - 28, 2020	May 8 - Jul 1, 2020	Jun 11, 2020 - Jan 15, 2021	Sep 16 - Dec 13, 2020
Modified intention-to-treat population	41	1033	1525	289
N. Patients in the treatment arm	**21**	**515**	**764**	**144**
Treatment arm	Ruxolitinib 5 mg bid	Baricitinib 4 mg per day for 14 days	Baricitinib 4 mg per day for 14 days	Tofacitinib 10 mg bid for 14 days
**PATIENT CHARACTERISTICS (treatment arm):**				
**(Median) Age (years old)**	**63**	(mean) **55**	(mean) **58**	(mean) **55**
**Male sex (%)**	**60**	**62**	**64**	**65**
Race/Ethnicity (%)	Asian 100	White 49 (Hispanic 25), Black 15, Asian 9.5, Other/Unknown 27	White 64 (Hispanic 21), Black 5, Asian 11, Other 20	White 82, Black 8, Mixed 8, Other/Unknown 2
(Median) BMI		(mean) 32	(mean) 30	29
Type-2 Diabetes (%)	25		29	24
Hypertension (%)	35		48	46.5
N. Days from hospitalization				5
N. Days from symptom onset		8	(mean) ≥7 days in 82%	10
**Glucocorticoids (%)**	**70**	**22.5**	**80**	**79**
Remdesivir (%)	0	100	18	0
Ambient air (%)	0	13.5	12	24
**LFO (%)**	**90**	**56**	**64**	**63**
**HFO or NIV/cPAP (%)**	**10**	**20**	**24**	**13**
IMV or ECMO (%)	0	10.5	0	0
**OUTCOMES (treatment vs. standard-of-care):**				
Clinical status or Functional Improvement (%)	**(14-day) 60 vs 43, p=NS;** **Time to improvement: 12 vs 15 days, p=NS**	**(15-day) OR 1.3** ***(HFO/NIV: OR 2.2)***		**Score worsening: OR 0.54**
(28-day) Discharge (%)	**Time to discharge: 17 vs 16 days, p=NS**	**Median time to recovery:** **7 vs 8 days, RR 1.16, p=0.03** ***(HFO/NIV: 10 vs 18 days, RR 1.51)***		**93 vs 89 (OR 1.05)**
(28-day) Progression (%)	**(14-day) Clinical deterioration:** **0 vs 19, p=NS;** **(14-day) CT improvement:** **90 vs 62, RR 1.45, p=0.0495**	**New use of oxygen:** **23 vs 40, HR 0.53**	**28 vs 30.5, p=NS, OR 0.85**	**Respiratory failure or death:** **18 vs 29, OR 0.63, p=0.04**
(28-day) IMV (%)		**10 vs 15, HR 0.64;** **IMV duration: 16 vs 27 days**		
(28-day) IMV or death (%)		**HR 0.69**		
**(28-day) Mortality (%)**	**0 vs 14, p=NS;** **cumulative incidence of death:** **RR 0.15, p=0.089**	**5 vs 8, HR 0.65;** **(*LFO: 2 vs 5, HR 0.40;*** ***HFO/NIV: 7.5 vs 13, HR 0.55)***	**8 vs 13, HR 0.57, p=0.0018** **(*HFO/NIV: 17.5 vs 29*,** ***HR 0.52, p=0.0065)***	**3 vs 5, HR 0.49**
Serious adverse events (%)	0 vs 19	**16 vs 21, p=0.03**	14 vs 18	14 vs 12
Serious infections (%)	0 vs 5	**6 vs 11, p=0.003** **(concomitant steroids: 25 vs 5.5)**	8.5 vs 10	3.5 vs 4
Venous thromboembolism (%)		4 vs 3	3 vs 2.5	1 vs 0
Effects on virus clearance	No impairment in IFNα production; **time to virus clearance 13 vs 12 days, p=NS**; **time to lymphocyte recovery: 5 vs 8 days, p=0.033; increased virus-specific IgM titers, p=0.039** [Cao et al. (107)]			

Differences in key characteristics compared to trials with IL-6 inhibitors are highlighted. LEGEND OF COLOURS in the CHARACTERISTICS session: Green: favourable characteristics in studies with IL-6 inhibitors; Blue: nearly-favourable characteristics in studies with IL-6 inhibitors; Red: unfavourable characteristics in studies with IL-6 inhibitors (see **Tables 1**, **2**).

LEGEND OF COLOURS in the OUTCOMES session: Green: results associated with proved efficacy and statistical significance; Blue: results associated with partial efficacy and numerical – but not statistical – differences; Red: non-significant results associated with lack of efficacy or adverse events.

HR, hazard ratio; OR, odds ratio; RR, relative risk; NS, not significant.

IL-1 inhibitors, in particular the recombinant IL-1α/β receptor antagonist anakinra, have been shown in observational studies to reduce mortality in both severe and critical disease (112, 113) (**Figure 3**). Whereas both IL-6 and IL-1 inhibitors would have a significant impact on mortality in patients showing a PaO2/FIO2 ratio ≥ 100 mmHg, only IL-1 inhibitors would remain effective in patients with a PaO2/FIO2 ratio < 100 mmHg (95). In fact, as previously mentioned, critical COVID-19 assumes the connotations of a hyperferritinemic syndrome or an autoinflammatory febrile disease, in which an aberrant activation of the NLRP3 inflammasome, in this case resulting from the conspicuous presence of monocytes-macrophages and viral products (e.g., viroporins), provokes intense production of IL-1α and IL-1β, which ultimately drive hyperinflammation and severe ARDS (7, 27), whereas IL-6 would now play regulatory roles. Two phase-3 RCTs evaluating the efficacy of IL-1 inhibitors in patients hospitalised with COVID-19 have recently been published, confirming significant beneficial effects on clinical status and survival for anakinra (98), but not for the anti-IL-1β monoclonal antibody canakinumab (114), thereby suggesting a central contribution of the alarmin IL-1α to acute lung inflammation and COVID-19 pathogenesis (7).

Limited data are available from RCTs in regard to therapies targeting other cytokines involved in severe COVID-19. Among GM-CSF inhibitors, otilimab (115) conferred a benefit on survival and respiratory status in a subgroup of patients aged ≥ 70 years old; lenzilumab (116) improved ventilator-free survival in hypoxic patients not receiving IMV; while for mavrilimumab (117) results were inconclusive, reporting numerical but not statistical differences on mortality, possibly due to the small size and the low power of the study. It also remains to adequately investigate the effects of inhibiting IL-17, by means of anti-IL-17A/F therapies (e.g., bimekizumab), in severe forms, or the alarmin cytokine IL-33, by means of anti-ST2 agents (e.g., astegolimab), at least in early and moderate stages, or in post-COVID fibrosis (7).

Regarding glucocorticoids, treatment with low-to-intermediate doses of steroids, particularly dexamethasone, has been shown to be associated with reduced mortality in hypoxic patients who require either IMV or supplemental oxygen, but not in those who do not require oxygen support (57, 80). Although the main RCT in this regard (57) did not distinguish between high-flow and low-flow oxygen requirements, other studies have reported non-significant effects of steroids in non-severe patients requiring only low-flow oxygen (118, 119) (**Figure 3**). In case of further disease progression despite glucocorticoid treatment, the addition of tocilizumab led to significantly better outcomes (120, 121), consistent with the synergistic effects of tocilizumab and dexametasone observed in the RCTs and meta-analyses discussed above (2, 58), and possibly explained by the synergism of IL-6R signal blockade combined with steroid-induced down-regulation of IL-6 (or other cytokines).

Since patients with COVID-19 at high risk of progression are characterised by early interferonopathy and viral replication (122), recombinant type-I (IFNβ) and especially type-III (IFNλ) interferons, as well as endosomal TLR agonists inducing IFNs, have been proposed in patients with mild-moderate disease and high viral load (7, 123–125), similarly to what was suggested in regard to antivirals (remdesivir) (126). Nevertheless, it has been argued that antivirals might help even in more advanced stages of the disease in combination with anti-inflammatory drugs (81, 126) (**Figure 3**).

COVID-19 is also characterised by acquired thrombophilia, and hypercoagulability might concur with hyperinflammation as a cause of death. Increased rates of venous thromboembolism have been observed in COVID-19 patients and autopsy studies demonstrated microvascular thrombosis in about 80% of cases (127). Furthermore, heparan sulfate may mediate virus interaction with host membrane receptors, thus promoting virus entry and inflammatory cell activation (7, 128). Heparin has been included in the treatment of COVID-19 with the double rationale of impeding clot formation and competing with membrane heparan sulfate. On the other side, hospitalisation could contribute to the observed risk of venous thromboembolism and the actual impact of pulmonary embolism and/or microvascular thrombosis on severe respiratory failure and mortality in COVID-19 is still controversial (127). Intensified anticoagulant strategies can halve the risk of venous thromboembolism but can also double the risk of bleeding (76), especially in critically-ill patients, possibly due to tissue hypoperfusion, concomitant supportive therapy with high-dose hydrocortisone, liver failure and coagulopathy. The question of whether to prefer anticoagulant therapy or prophylaxis is therefore the subject of intense debate. Plasma concentrations of D-dimer might be used to guide anticoagulation dose in hospitalised patients (97). Presumably, D-dimer levels < 750-1000 ng/mL (moderate disease) may suggest a low-dose prophylaxis; levels between 1000 and 3000 ng/mL (severe disease) may suggest intermediate- or high-dose therapeutic regimens; while levels > 3000-5000 ng/mL (critical disease) may warn of the risk of bleeding with intensified strategies (**Figure 3**). In fact, a recent meta-analysis seems to show that a full-dose anticoagulation may reduce mortality in severe, yet non-critical, patients (76).

Further data from ongoing or recently completed clinical trials are eagerly awaited (**Table 4**). In this regard, it will be particularly interesting to assess the effect of combining IL-6 inhibitors with antivirals (e.g., tocilizumab + remdesivir, tocilizumab + favipiravir), anticoagulants (e.g., tocilizumab + full-dose heparin), IL-1 inhibitors (e.g., tocilizumab + anakinra, tocilizumab + colchicine), or other immunotherapies (e.g., tocilizumab + pembrolizumab). Ongoing head-to-head comparative studies (e.g., tocilizumab ± remdesivir versus baricitinib ± remdesivir) are of great interest as well.

**Table 4:** Randomized controlled clinical trials with agents targeting the IL-6 signalling in COVID-19 that are ongoing or awaiting results (as reported in *Clinicaltrials.gov*; last accessed December 6, 2021).

Trial ID	Name	Phase	Masking	Recruitment Status	Actual (or Estimated) Completion Date	Actual (or Estimated) Number of Patients Enrolled	(Main)Location	Experimental Intervention(s)	Comparator(s)
NCT04479358	COVIDOSE-2	II	open-label	recruiting	March 2023	(332)	USA	low-dose tocilizumab (40 or 120 mg)	standard-dose tocilizumab or SOC
NCT04412772	ARCHITECTS	III	double-blind	recruiting	December 2021	(300)	USA	tocilizumab	placebo
NCT04377750		IV	open-label	recruiting	(May 2021)	(500)	Israel	tocilizumab	placebo
NCT05002517		III	triple-blind	active, not recruiting	(October 2021)	60	Spain	tocilizumab	methylprednisolone
NCT04519385		N/A	double-blind	**completed**	August 2020	69	Egypt	tocilizumab	dexamethasone
NCT04476979	TOCIDEX	II	open-label	recruiting	December 2021	(660)	French Guiana	tozilizumab + dexamethasone	dexamethasone
NCT04577534	COVIDSTORM	III	open-label	**completed**	June 2021	88	Finland	tocilizumab	SOC
NCT04690920		N/A	open-label	**completed**	December 2020	**200**	Pakistan	tocilizumab or remdesivir	SOC
NCT04412291	ImmCoVA	II	open-label	recruiting	(June 2021)	(120)	Sweden	tocilizumab or anakinra (+ SOC)	SOC
NCT04678739		III	open-label	**completed**	February 2021	**205**	Bangladesh	**tozilizumab + remdesivir**	SOC
NCT04409262	REMDACTA	III	double-blind	**completed**	March 2021	**649**	USA	**tocilizumab + remdesivir**	placebo + remdesivir
NCT04779047		IV	open-label	recruiting	(April 2021)	(150)	Egypt	**tocilizumab + remdesivir** + lopinavir/ritonavir	**tocilizumab + ivermectin** + hydroxychloroquine
NCT04310228		N/A	open-label	recruiting	(May 2020)	(150)	China	**tocilizumab + favipiravir**	tocilizumab or favipiravir
NCT04424056		III	open-label	not yet recruiting	November 2022	(216)	France	**tocilizumab** or anakinra **± ruxolitinib**	SOC
NCT04330638	COV-AID	III	open-label	**completed**	April 2021	**342**	Belgium	tocilizumab or siltuximab or anakinra or **tocilizumab + anakinra** or siltuximab + anakinra	SOC
NCT04600141	HEPMAB	III	open-label	recruiting	December 2021	(308)	Brazil	**tocilizumab + heparin** (**therapeutic or prophylactic** anticoagulation with **UFH or LMWH**)	heparin (therapeutic or prophylactic, UFH or LMWH)
NCT05118737		I	open-label	recruiting	August 2022	(230)	Qatar	**tocilizumab + colchicine**	tocilizumab
NCT04335305	COPERNICO	II	open-label	recruiting	(June 2021)	(24)	Spain	**tocilizumab + pembrolizumab**	SOC
NCT04347031		II/III	open-label	**completed**	November 2020	**320**	Russia	**mefloquine** or hydroxychloroquine + azithromycin **± tocilizumab**	mefloquine or hydroxychloroquine
NCT04346693		III	open-label	**completed**	November 2020	**320**	Russia	**leitragin** (intramuscular or inhaled) + hydroxychloroquine + azithromycin **± tocilizumab**	hydroxychloroquine + azithromycin ± tocilizumab
NCT04357860	SARICOR	II	open-label	**completed**	April 2021	120	Spain	sarilumab sc (200 or 400 mg) (+ SOC)	SOC
NCT04357808	SARCOVID	II	open-label	**completed**	December 2020	30	Spain	sarilumab sc (+ SOC)	SOC
NCT04359901		II	open-label	active, not recruiting	April 2023	50	USA	sarilumab sc (+ SOC)	SOC
NCT04324073	CORIMUNO-SARI	II/III	open-label	active, not recruiting	December 2021	239	France	sarilumab	SOC
NCT04329650		II	open-label	recruiting	(May 2020)	(200)	Spain	siltuximab	methylprednisolone
NCT04343989		II	double-blind	**completed**	March 2021	180	USA	**clazakizumab** (25 mg or 12.5 mg)	placebo
NCT04363502		II	triple-blind	recruiting	May 2022	(30)	USA	clazakizumab	placebo
NCT04494724		II	quadruple-blind	recruiting	(July 2021)	(60)	USA	clazakizumab	placebo
NCT04380961		II	quadruple-blind	**completed**	June 2021	**212**	USA	**sirukumab** (+ SOC)	placebo (+ SOC)
NCT04380519		II/III	double-blind	**completed**	July 2020	**372**	Russia	**olokizumab** or RPH-104/goflikicept (+ SOC)	placebo (+ SOC)
NCT05056558		III	triple-blind	not yet recruiting	September 2022	(480)	Bangladesh	baricitinib (+ SOC)	placebo (+ SOC)
NCT04891133	EU SolidAct	II/III	quadruple-blind	recruiting	September 2025	(1900)	Austria	baricitinib (+ SOC)	placebo (+ SOC)
NCT04346147	Covid19 COVINIB	II	open-label	active, not recruiting	(September 2021)	168	Spain	baricitinib or imatinib	supportive treatment
NCT04390464	TACTIC-R	IV	open-label	recruiting	May 2022	(1167)	UK	baricitinib or ravulizumab (+ SOC)	SOC
NCT04970719		III	open-label	recruiting	December 2021	(382)	Bangladesh	baricitinib + remdesivir	**dexamethasone + remdesivir**
NCT04832880	AMMURAVID	III	open-label	not yet recruiting	December 2022	(4000)	Italy	baricitinib + remdesivir + dexamethasone or baricitinib + dexamethasone or **remdesivir + dexamethasone**	dexamethasone
NCT04640168	ACTT-4	III	double-blind	**completed**	June 2021	**4074**	USA	baricitinib + remdesivir + placebo	placebo + **remdesivir + dexamethasone**
NCT04403243	COLORIT	II	open-label	recruiting	(August 2020)	(70)	Russia	ruxolitinib or colchicine or secukinumab	SOC
NCT04581954	MATIS	I/II	single-blind	recruiting	December 2021	(456)	UK	ruxolitinib or fostamatinib	SOC
NCT04348695	Ruxo-Sim-20	II	open-label	recruiting	(May 2020)	(94)	Spain	**ruxolitinib + simvastatin**	SOC
NCT05082714		N/A	open-label	recruiting	April 2022	(164)	Greece	**tocilizumab**	**baricitinib**
NCT04693026		III	open-label	recruiting	(March 2021)	(150)	Bangladesh	**tocilizumab + remdesivir**	**baricitinib + remdesivir**

SOC, standard-of-care; UFH, unfractionated heparin; LMWH, low-molecular-weight heparin.

Bold text indicates completed studies awaiting results with a relevant number of participants or testing additional IL-6 inhibitors, interesting combinations or comparations.

## Discussion and Conclusions

Severe COVID-19 is a hyperinflammatory and life-threatening pulmonary and systemic disease, so prompt intervention on the cytokine cascade can importantly prevent clinical deterioration and mortality.

The beneficial effects of tocilizumab and other biologics in the management of COVID-19 have long been debated owing to large discrepancies in study results, possibly due to differences in sample size (i.e., studies often underpowered to detect significant differences in mortality), patient series composition (i.e., disease severity, magnitude of systemic inflammation, age, comorbidities, and perhaps sex and ethnicity), and treatment protocols (i.e., dosage, timing and co-treatments, particularly with steroids).

Here, we attempted to dissect the heterogeneity of these studies as well as the complexity of the inflammatory cascade in COVID-19, the differential roles of IL-6 in relation to disease stage and severity, and the biological significance of clinical parameters and laboratory markers, in order to identify and summarize the baseline characteristics of patients who should best respond to treatment with IL-6 inhibitors or other biologics, thus ultimately defining precise therapeutic windows.

Therapeutic approaches for COVID-19 based on disease stage have been suggested by subgroup analyses of clinical trials and in part summarized in previous editorials (129). Our review extends therapeutic options to anti-cytokine monoclonal antibodies and decoy receptors, such as IL-6, GM-CSF, and IL-1 inhibitors, in addition to JAK inhibitors, antivirals and glucocorticoids, with the ultimate aim of highlighting their differential windows of opportunity. Importantly, our estimation of disease severity is based not only on respiratory status, here assessed by means of the PaO2/FIO2 ratio and oxygen requirement, but also on key circulating biomarkers, namely IL-6, CRP, ferritin, D-dimer and LDH levels. Furthermore, here we have distinguished a “severe-to-critical” stage corresponding to early ICU patients, including those undergoing IMV within the past 24 hours (i.e., ordinal score between 6 and 7 on the NIAID 8-point ordinal scale for assessing clinical status), for whom – so far - there is evidence of benefit for IL-6 inhibitors, but not for JAK inhibitors.

In summary, due to the pathogenic role of IL-6 signal (trans-signalling and trans-presentation) specifically in the severe stage of the disease, IL-6 inhibitors, and particularly anti-IL-6R monoclonal antibodies (e.g., tocilizumab, sarilumab), appear to be effective in patients with severe COVID-19, mostly characterised by baseline IL-6 levels between 35 and 90 ng/mL (typically reached within 6 days of hospitalisation), PaO2/FIO2 ratios between 100 and 200 mmHg, requirement of HFO or NIV, CRP levels between 120 and 160 mg/L, ferritin levels between 800 and 1600 ng/mL, D-dimer levels between 750 and 3000 ng/mL, and LDH levels between 350 and 500 U/L. Patients aged between 59 and 64 years old, males, and non-Hispanic White, might respond better than others to IL-6 inhibitors.

Because GM-CSF also plays an important role in severe disease, GM-CSF inhibitors (e.g., lenzilumab, otilimab, mavrilimumab) may have a therapeutic window similar to that of IL-6 inhibitors, perhaps with a difference in age preference (i.e., better outcomes obtained for patients aged ≤ 70 years old with tocilizumab, ≥ 70 years old with otilimab).

Since JAK1 and JAK2 transmit both IL-6 and GM-CSF signals, as well as the signal of various cytokines placed aside or upstream in the inflammatory cascade (e.g., IL-9, IFNγ, IL-7, IL-23, G-CSF), JAK inhibitors, and particularly JAK1/JAK2 inhibitors (e.g., baricitinib, which might also directly interfere with viral replication), appear to be effective not only in severe forms but also in moderate COVID-19 (thus including younger patients and more women), and even in the absence of concomitant steroids.

Whereas IL-6 acquires homeostatic roles in critical stages (cis-signalling), IL-1α and IL-1β are instead proinflammatory and pathogenic in both severe and critical disease, being increasingly released with severe hyperferritinemia and hyperactivation of monocytes-macrophages; IL-1 inhibitors, in fact, and particularly the decoy IL-1 receptor binding to both IL-1 isoforms (e.g., anakinra), appear to be effective not only in moderate-to-severe forms but also in critical COVID-19.

It is hoped that this narrative overview of the current literature may offer useful insights into the proper use of biologics in COVID-19, in future trials as in the real world.

## Author Contributions

GZ conceived the study, searched the literature, wrote the manuscript, and prepared the figures and tables. AT, LC, and AL contributed to search the literature and reviewed the manuscript. NM, PF, IS, and AM critically edited the manuscript. All authors have read and approved the final version submitted.

## Conflict of Interest

The authors declare that the research was conducted in the absence of any commercial or financial relationships that could be construed as a potential conflict of interest.

## Publisher’s Note

All claims expressed in this article are solely those of the authors and do not necessarily represent those of their affiliated organizations, or those of the publisher, the editors and the reviewers. Any product that may be evaluated in this article, or claim that may be made by its manufacturer, is not guaranteed or endorsed by the publisher.

## Abbreviations

ACE2, angiotensin-converting enzyme 2; ARDS, acute respiratory distress syndrome; CAR-T, chimeric antigen receptor T-cell therapy; CCL, C-C motif chemokine ligand; CD, cluster of differentiation; cPAP, continuous positive airway pressure; CXCL, C-X-C motif chemokine ligand; COVID-19, Coronavirus Disease 2019; CRP, C-reactive protein; CRS, cytokine release syndrome; DAMPs, damage-associated molecular patterns; ECMO, extracorporeal membrane oxygenation; FABP4, fatty acid-binding protein 4; Fc, fragment crystallisable; FCN1, ficolin-1; G-CSF, granulocyte colony-stimulating factor; GM-CSF, granulocyte-macrophage colony-stimulating factor; gp130, 130-kDa glycoprotein; HFO, high-flow oxygen; i, inhibitors; HMGB1, high mobility group box-1; ICU, intensive care unit; IFN, interferon; IL, interleukin; IMV, invasive mechanical ventilation; JAK, Janus kinase; LDH, lactate dehydrogenase; LFO, low-flow oxygen; M1 or M2, type-I or type-II (classically or alternatively) activated macrophages; mAbs, monoclonal antibodies; MAS, macrophage activation syndrome; NETs, neutrophil extracellular traps; NIAID, National Institute of Allergy and Infectious Diseases; NIH, National Institutes of Health; NIV, non-invasive ventilation; NLRP3, NLR family pyrin domain containing 3; PAMPs, pathogen-associated molecular patterns; PaO2/FIO2 ratio, pressure of arterial oxygen to fractional inspired oxygen concentration; -R, receptor; -RA, receptor antagonist; RCT, randomised controlled trial; RORC, retinoic acid orphan receptor C; s-, soluble; SaO2, peripheral arterial oxygen saturation; SARS-CoV-2, severe acute respiratory syndrome coronavirus 2; SOCS, suppressor of cytokine signalling; SPP1, secreted phosphoprotein 1; STAT, signal transducer and activator of transcription; suPAR, soluble urokinase plasminogen activator receptor; TH, T helper; TH-GM, GM-CSF-producing T helper cells; TLR, Toll-like receptor; TNF, tumor necrosis factor; Treg, regulatory T cells.
